# Supplementary figures and images for: From birth to adulthood: An analysis of the Brazilian lancehead (Bothrops moojeni) venom at different life stages
Source: PLoS One. 2021 Jun 10;16(6):e0253050. doi: 10.1371/journal.pone.0253050 (PMC8191990; doi:10.1371/journal.pone.0253050)

**Fig 2.**

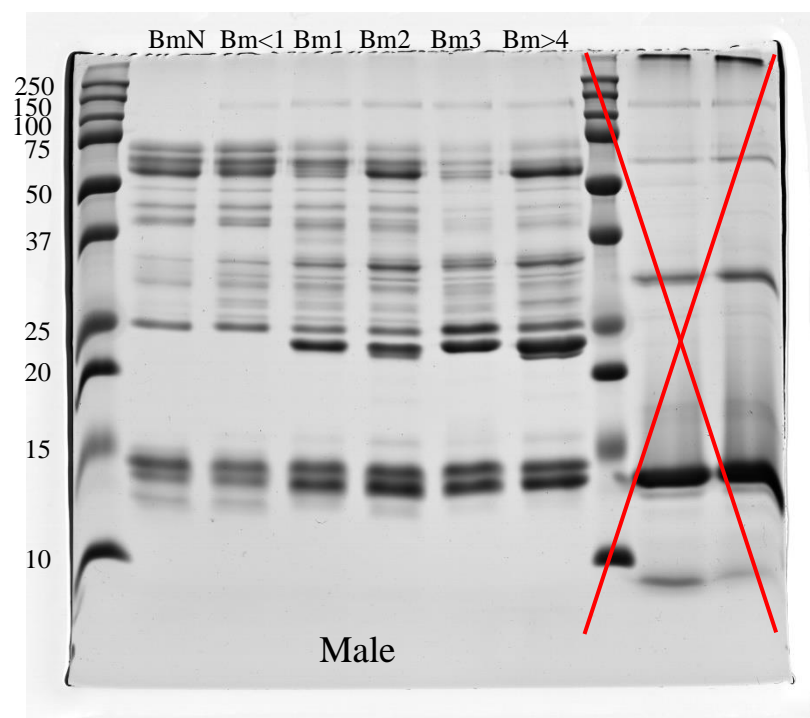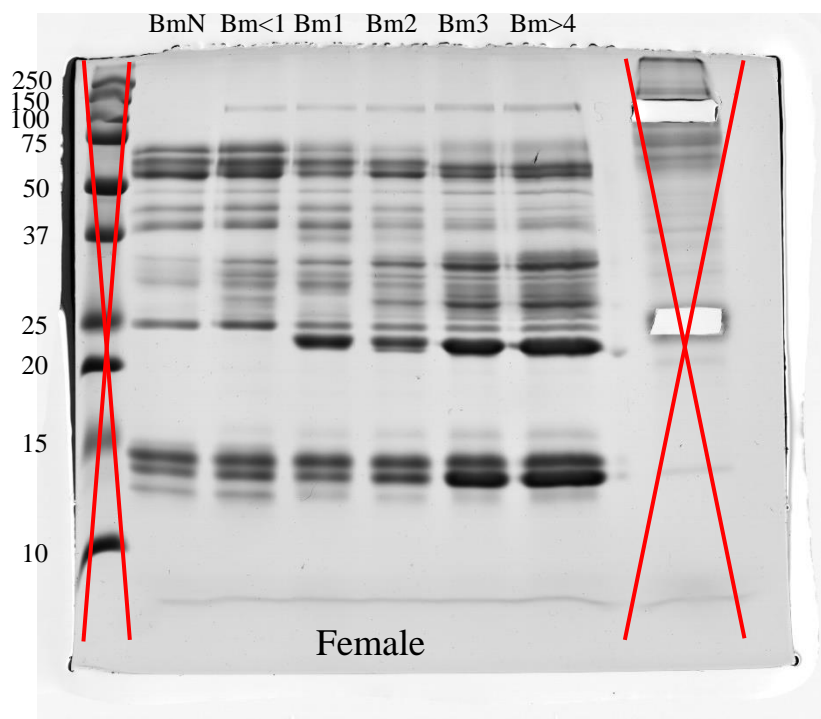

**Fig 6.**

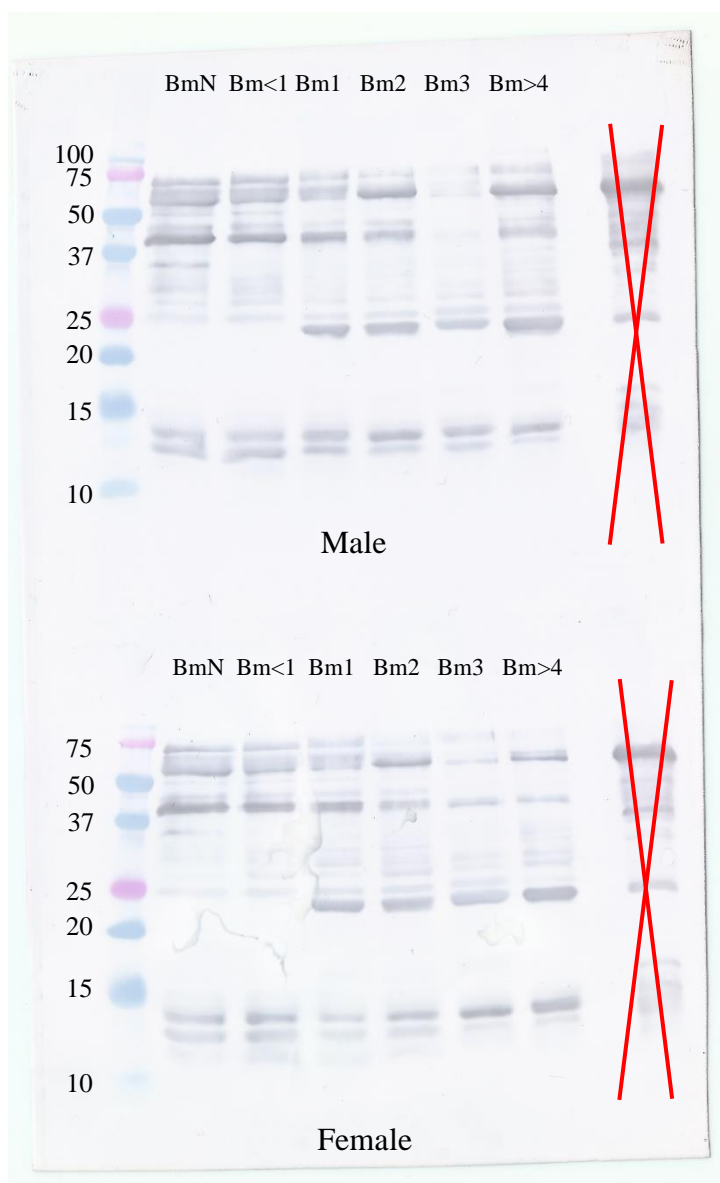

Supplement: S1 Raw images — (PDF) [file pone.0253050.s001.pdf]
