# Supplementary material for: From birth to adulthood: An analysis of the Brazilian lancehead (Bothrops moojeni) venom at different life stages
Source: PLoS One. 2021 Jun 10;16(6):e0253050. doi: 10.1371/journal.pone.0253050 (PMC8191990; doi:10.1371/journal.pone.0253050)
Supplement: Attachment — Submitted filename: Response to Reviewers.docx [file pone.0253050.s006.docx]

**Response to Reviewers**

**Reviewer #1: Major comments**

1. Venoms from various ontogenetic stages were collected from snakes belonging to distinct geographical regions. How can the authors be sure that the differences they see are because of ontogenetic shifts, and not because of differences in geographical locations (i.e., intraspecific / interpopulation variation)? Wouldn’t it make much more sense to compare snakes of different ontogenetic stages from the same locality?

We would like to thank the reviewer for this inquiry. We understand the concern and we are aware of this issue. Indeed, in an ideal situation, it would be much better to compare the venoms of snakes from the same locality. However, we did not dispose of such material. Furthermore, a previous work by our group (Aguiar et al. Comparative compositional and functional analyses of *Bothrops moojeni* specimens reveal several individual variations; PloS ONE 14(9): e0222206, 2019) compared individual venoms of 13 snakes from different localities but did not identified a relationship between activities and geographical location. We understand the limitations of working with pooled venoms, but it is what we had available.

2. Male and Female venoms at different stages are represented by unequal sample sizes. Could the effect of unequal pooling influence the overall functional differences?

We thank the reviewer for pointing this out. This difference may have influenced the outcome of some results. However, we collected the venoms from the snakes we had available and we described this limitation in the Discussion (lines 588-600).

3. Authors should describe their relative quantification steps in detail. LFQ works best when the spectra are searched against species-specific transcriptomes or when working with model organisms. Performing searches against the Uniprot database, that too by restricting it to a specific clade, is likely to produce erroneous results.

Thank you for pointing this out. We add more details of the LFQ. We used the three most intense peptide ions to compare the relative abundances of the quantified proteins. We agree with the reviewer that LFQ would work best with species-specific databases. However, the aim of our quantitative approach was only to estimate and compare proteins at the protein family level.

4. The RAW mass spec data should be made available to the readers. Authors should consider depositing this to public repositories, such as the ProteomeXchange

The proteomics data have been deposited to the ProteomeXchange Consortium via the PRIDE partner repository with the dataset identifier PXD024447.

Submission details:

Project Name: From birth to adulthood: An analysis of Bothrops moojeni snake venom at different life stages

Project accession: PXD024447

Project DOI: Not applicable

Reviewer account details:

Username: reviewer_pxd024447@ebi.ac.uk

Password: KjgIbK2q

Minor comments

1. Protein concentrations of different samples should be presented in table 1.

We thank the reviewer for this suggestion. We added this information to Table 1.

2. Line 423 could be Neonate and/or Juvenile snake as haemorrhagic activity was not tested for juveniles in this study.

Thank you for the suggestion. We replaced “juvenile or an adult” by “neonate, juvenile, or adult” (line 477).

3. “B. moojeni snake venoms were milked at the Laboratory of Herpetology of Instituto Butantan, according to the laboratory protocol” The authors should clarify what lab protocol they are referring to here.

We thank the reviewer for the suggestion. The article describing the Laboratory of Herpetology protocol was added (line 121).

4. Table 1 is very confusing. It might make it clear to separate the number of individuals from the SVN data.

Thank you for the suggestion. We separated number of individual from the average size of the snakes.

5. In the materials and methods, the authors are advised to provide catalogue numbers of the chemicals that they have used, which will make it easier for the readers to reproduce these results.

Thank you for pointing this out. We added this information in Materials and Methods.

Grammatical errors

The manuscript has many grammatical errors. I have only listed a few below.

1. Line 31: “in specific groups”

2. Suggest rewriting this awkward sentence: “has been studied for many years, as well as individual variation”. Moreover, I fail to understand this point as the individual venom variation is driven by the other reasons they mention in this sentence.

3. “their respective offspring of nine species of Bothrops”

4. “in regards of clinical outcome”

5. “have an impact in snakebite treatment”

6. “until the analyses Table 1 shows the groups, ages, number of”

7. “According to previous works [20,22,63–67], protein families were assigned.”

We would like to thank the reviewer for all these suggestions. We revised the manuscript and corrected the mistakes.

**Reviewer #2:**

The experimental methodology (assays, LC-MS/MS, SDS-PAGE, etc.) evaluating the venoms of Bothrops moojeni from birth to adulthood is robust, as is the discussion incorporating results from other publications. My major concern is that the pools of venom from different age groups were from snakes of different localities. Therefore, some of the variation trends could be due to geographic variation and not ontogeny. This manuscript might also be better suited in a journal with more specific readership, such as the journal Toxicon.

We would like to thank the reviewer for this inquiry. We understand the concern and we are aware of this issue. Indeed, in an ideal situation, it would be much better to compare the venoms of snakes from the same locality. However, we did not dispose of such material. Furthermore, a previous work by our group (Aguiar et al. Comparative compositional and functional analyses of *Bothrops moojeni* specimens reveal several individual variations; PloS ONE 14(9): e0222206, 2019) compared individually the venoms of 13 snakes from different localities but did not identified a relationship between activities and geographical location. We understand the limitations of working with pooled venoms, but it is what we had available.

Minor suggestions for improvement:

Abstract
I feel an additional sentence should be added about the 3 years-old snake venoms, were they similar to juveniles or different from both juveniles and adults? This detail is missing from the abstract currently.

Thank you for the suggestion. We included a sentence about the 3 years-old snake venoms (lines 32-33).

Introduction
The second paragraph is a little short. It would be important to mention what toxins specifically are in this venom and what are the activities of the major toxins (metalloproteinases, serine proteases, etc.).

We would like to thank the reviewer for the suggestion. We included this information in the Introduction (lines 55-66).

Line 68: More detail should be added here, such as how was the venom different between the adult females and offspring (what components specifically)?

Thank you for the suggestion. We rewrote this part to include this information (lines 90-94).

Material and methods

Any statistics that could also incorporate geographical variation?

We appreciate the suggestion. However, due to sample shortage (especially of younger snakes), the analyses were carried out using pooled venoms, so we do not have specific data of each individual activity or composition. Therefore, we agree with the reviewer that this information could improve our work, but it is not possible to add it.

Results
Figure 2. It would be nice to see the protein families labeled on the side of the gel. Also, arrows pointing out the differences are that are discussed.

We would like to thank the reviewer for this suggestion. We inserted the protein families to the image.

Figure 3. Figure resolution needs to be improved. I cannot see the labels on the chromatograms, including the x- and y-axis, they should be larger and clearer. What does the asterisk signify? This should be noted in the legend.

Thank you for pointing this out. We separated this figure in two parts so it can be easier to see and fit into the page. We also included the information regarding the asterisk.

Line 342: “greatest” concentration

We corrected this mistake.

Figure 5: None of these were statistically significant? If so, an asterisk should be added to the corresponding comparison.

We thank the reviewer for this inquiry. Actually, many groups present statistical differences among each other. To avoid confusion and to keep the image cleaner, we decided to include this information in the Supplementary Table 2.

Figure 7. Like the other gel, it would be nice to see the protein families labeled on the side of the gel near their size ranges.

We would like to thank the reviewer for this suggestion. We inserted the protein families to the image.

Discussion
Line 427: A different word then “besides” should be used. Perhaps begin this sentence listing the authors of the publication?

Thank you for the suggestion. We modified the sentence from “Besides, the appearance of a band weighing ~23 kDa and the change of low molecular weight proteins were shown in *B. atrox* venom” to “López-Lozano and collaborators observed the appearing of a ~23 kDa band and the change of low molecular weight proteins in *B. atrox* venom” (lines 482-484).

The discussion is a bit long.

We thank the reviewer for the concern. However, we believe that the discussion contains the necessary information to corroborate the results and the importance of our work.
