# Supplementary material for: From birth to adulthood: An analysis of the Brazilian lancehead (Bothrops moojeni) venom at different life stages
Source: PLoS One. 2021 Jun 10;16(6):e0253050. doi: 10.1371/journal.pone.0253050 (PMC8191990; doi:10.1371/journal.pone.0253050)
Supplement: Attachment — Submitted filename: Response to Reviewers.docx [file pone.0253050.s007.docx]

**Reviewer #1:** Authors still need to address the following major comments

“but it is what we had available”

I don’ think this is an appropriate response. The authors should acknowledge this shortcoming very clearly in their manuscript.

“However, we collected the venoms from the snakes we had available and we described this limitation in the Discussion (lines 588-600).”

Those lines do not contain any information related to this. Authors need to very clearly explain the shortcomings of this study. Perhaps, in a different section. I am sympathetic to their situation and I do realize its not very easy to do these under ideal conditions. But there is no excuse for not highlighting the obvious shortcomings of the study clearly. This will help the readers judge the results and carve out future directions for research.

We thank the reviewer for this observation. We were not very clear about this shortcoming, so we tried to clarify this issue in the manuscript (lines 575-580).

Protein quantification

This should also be perhaps highlighted in the drawbacks section. Pleas see reviews by Calvete et al. The fact that LFQ was performed can be ignored as long as this is clearly stated as one of the drawbacks. However, restricting searches against Bothrops is just technically wrong. Please check how many Bothrops sequences are there on Uniprot compared to overall number of sequences. Only the reviewed Bothrops sequences are included on Uniprot, which means that by restricting your searches to these handful of sequences, you are increasing your chances of missing out on a much larger number of hits. Uniprot itself is such a small database so there is no excuse for not searching against the complete database.

Thank you for the comment. The reviewer is correct, we have less toxins restricting the database to the reviewed Bothrops sequences. However, as the objective of this database search was to classify toxins only at the family level (and not at the toxin level), we believe we have the main toxins represented in the reviewed database.

**Reviewer #2:** The revised manuscript has greatly improved. There is still the caveat of using pooled venom from snakes of different localities, but given at this time there is no way to fix this, the authors just need to make it very clear in the writing that this is a major limitation in this work and how the results could be impacted. The resolution of the figures still appears a little blurry, I would just make sure they are sharper for publication.

We thank the reviewer for this observation. We tried to clarify this issue regarding the samples in the manuscript (lines 575-580). As suggested before, we used PACE to optimize the images, but it seems some issues might not have been corrected. We reuploaded the images to PACE and hope this time this matter is resolved.

Some minor spelling/grammar issues are still present, I have pointed out a few of these to be corrected, but the manuscript should be edited again:

Abstract Line 39: “highlights the importance of comprehensive studies to better understand venom variability.”

Line 43: “health”

Line 54: inespecifically?

Line 60: “disrupts blood homeostasis” ? Or maybe a better term to clarify?

Line 65: How come only in some localities? Maybe some details to clarify, such as does venom variability for this species have noted variation in antivenom effectiveness? The way the sentence is currently written (and the first line of the abstract) it seems like that it is only a public health problem in some areas and in others it is harmless.

Line 94: “change” instead of modifies

Fig 3 and Fig 4 titles: change to “male B. moojeni” and “female B. moojeni”
Similar title change for Fig 5 and Fig 7. It is best to use B. moojeni throughout the manuscript to avoid nonspecific common names.

Fig 7. “for”? typo

Lines 584: I would clarify this sentence a bit more. The word “promoter” is associated with gene transcription, and miRNAs are post-transcriptional regulators. miRNAs do not necessarily regulate genes, but the translation of their mRNAs.

We would like to thank the reviewer for all the suggestions. We modified the manuscript accordingly.
